# CTLA4 overexpressing adipose tissue-derived mesenchymal stem cell therapy in a dog with steroid-refractory pemphigus foliaceus

**DOI:** 10.1186/s12917-015-0371-3

**Published:** 2015-03-06

**Authors:** Sei-Myoung Han, Hyun-Tae Kim, Kun-Woo Kim, Kee-Ok Jeon, Kyoung-Won Seo, Eun Wha Choi, Hwa-Young Youn

**Affiliations:** Department of Veterinary Internal Medicine, College of Veterinary Medicine, Seoul National University, Seoul, 151-742 Republic of Korea; Department of Veterinary Internal Medicine, College of Veterinary Medicine, Chungnam National University, Daejeon, 305-764 Republic of Korea; Laboratory Animal Research Center, Samsung Biomedical Research Institute/ School of Medicine, Sungkyunkwan University, 81 Irwon-ro, Gangnam-gu, Seoul 135-710 Republic of Korea

**Keywords:** Autoimmune disease, CTLA4, Dog, Mesenchymal stem cell, Pemphigus foliaceus

## Abstract

**Background:**

Canine pemphigus foliaceus is an autoimmune antibody-mediated skin disease characterized by acantholysis. The objective of this case report is to present the successful management of steroid refractory pemphigus foliaceus with cytotoxic T-lymphocyte antigen 4 (*CTLA4*)-overexpressing adipose tissue mesenchymal stem cells (ATMSCs).

**Case presentation:**

A 10-year-old, 12.3-kg, castrated male Shih Tzu presented with severe pruritus and anorexia. The diagnosis of pemphigus foliaceus was made based on its history, physical examination, and histopathology results of a skin biopsy. Treatment with prednisolone and combination therapy of other immunosuppressive drugs had failed; therefore, immunosuppressive gene, CTLA4 overexpressing ATMSCs (CTLA4-ATMSCs) and/or naive ATMSCs administration was performed with the consent of the owner. ATMSCs were administered 21 times over a period of 20 months with intervals of 2 to 8 week. Prednisolone was gradually tapered concurrently and no relapse of the clinical signs was observed. After the termination of CTLA4-ATMSCs and/or naive ATMSCs treatment, the skin lesions had improved and could be managed with a low dose of prednisolone for 12 months.

**Conclusion:**

CTLA4-ATMSCs or naive ATMSCs transplantation may be beneficial as adjunctive therapy to initiate and maintain the remission of skin lesions caused by pemphigus foliaceus in veterinary medicine.

## Background

Canine pemphigus foliaceus is an autoimmune antibody-mediated skin disease characterized by acantholysis. The pathogenesis involves the production of autoantibodies against a target protein in the adhesion molecules of keratinocytes [1]. Desmoglein I is the main antigen implicated in pemphigus foliaceus in dogs and humans [2,3]. Binding of antibodies to adhesion molecules such as Desmoglein I disrupts the intercellular cohesion of keratinocytes. This results in acantholysis and the typical lesions seen in pemphigus, including formation of blisters and intra-epidermal pustules. The cause is usually unknown. However, some cases are possibly drug-induced [4] or a sequel to a chronic inflammatory skin disease [5]. The most successful treatment for canine pemphigus foliaceus is immunosuppression [6] with corticosteroids or cyclosporine. In recent studies, however, side effects of this treatment such as diarrhea, polyuria/polydipsia, weight gain, and recurrent infections have been described [7,8]. Moreover, it has been reported that only 53% of treated cases survive for more than 1 year after initiation of treatment [8].

Mesenchymal stem cells (MSCs) have immunosuppressive properties and inhibit a variety of cell types that mediate both the adaptive and the innate immune response [9,10]. MSCs suppress CD4+ and CD8+ T lymphocytes independently and appear to inhibit the differentiation of and antibody production by B cells [11,12] as well as the activation and expansion of natural killer (NK) cells [13]. In addition, MSCs have the ability to modulate T-cell proliferation and function. Based on these abilities, MSCs have been proposed as a therapeutic option in the treatment of autoimmune diseases and related gene overexpression shows an increased therapeutic effect in autoimmune diseases [14]. In our previous study, we tested the supernatant from adipose tissue-derived MSCs (ATMSCs) and cytotoxic T-lymphocyte antigen 4 (*CTLA4*)-overexpressing ATMSCs (CTLA4-ATMSCs) for suppressive effects on the proliferation of peripheral blood mononuclear cells (PBMCs) from dogs with experimental autoimmune thyroiditis. Addition of the supernatant from CTLA4-ATMSCs suppressed the proliferation of PBMCs stimulated with thyroglobulin (autoantigen), when compared to the addition of supernatant from ATMSCs. Under the same controlled conditions, CTLA4-ATMSCs showed a stronger suppression than nontransduced ATMSCs on the proliferation of the autoantigen [15]. Therefore, CTLA4, which has an inhibitory effect on T-cells, was transduced into ATMSCs to increase the immunosuppressive ability.

This case report describes the successful management of steroid refractory pemphigus foliaceus with CTLA4-ATMSCs and/or naive ATMSCs. The treatment resulted in the remission of the clinical signs that could be managed with a low dose of prednisolone. No recurrence was observed for 12 months.

## Case presentation

A 10-year-old, 12.3 kg, castrated male Shih Tzu was referred to the Veterinary Medicine Teaching Hospital (VMTH) of Seoul National University with severe pruritus and anorexia for the past 7 months. The dog had been treated with prednisolone orally twice daily (4.4 mg/kg/day) and systemic antibiotics after the diagnosis of pemphigus foliaceus was made at a local veterinary clinic. Despite the treatment, the waxing and waning lesions worsened over time.

**Figure 1:**
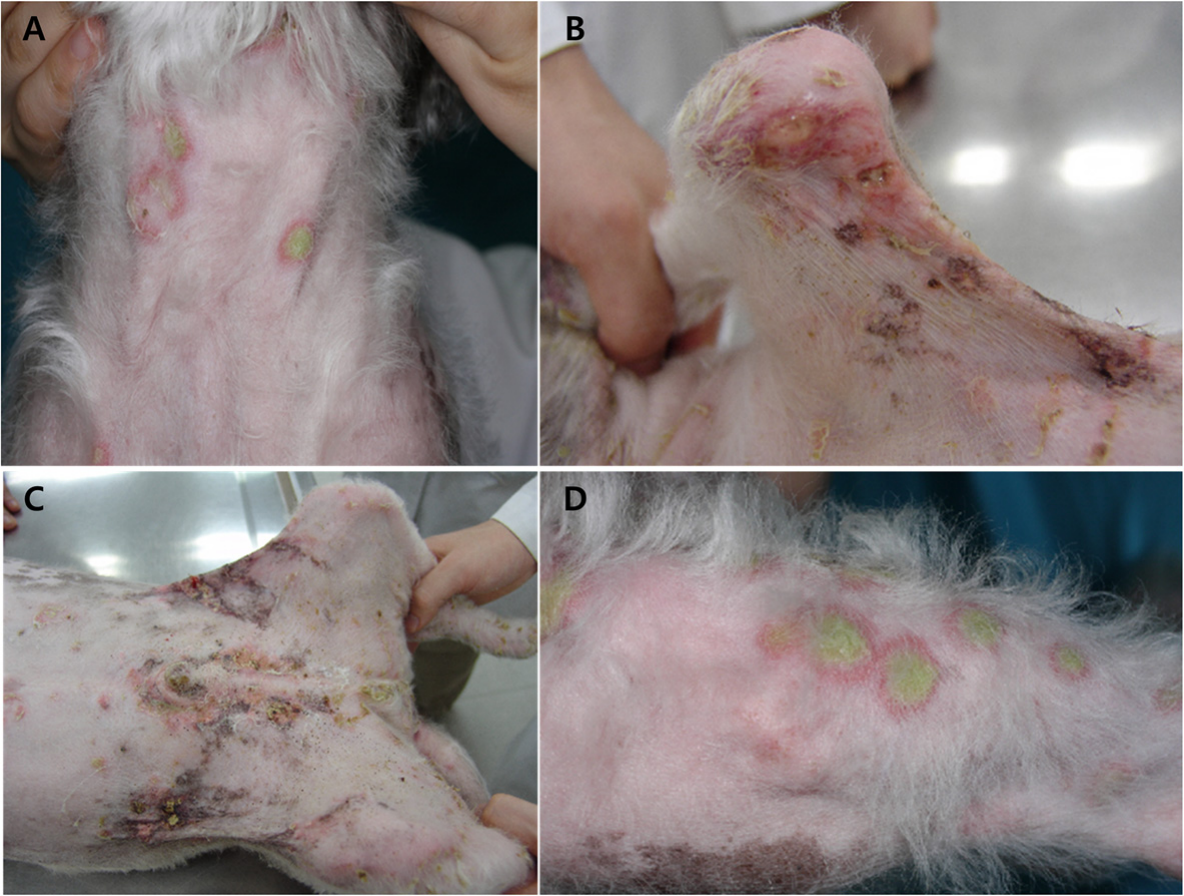
**Initial physical examination.** Erythema, papules, pustules, crusts, lichenification, and erosions on the neck **(A)**, elbow and axilla **(B)**, abdomen and inguinal area **(C)**, and the dorsal region of the trunk **(D)**.

Physical examination revealed mild depression and generalized crusting. Papules and pustules were seen on the neck, elbows, ears, abdomen, perianal area, inguinal area, axillae, and the dorsal part of the trunk. Alopecia of the forelimbs was also evident (Figure 1). Skin scrapings were performed to exclude *Demodex canis* and other ectoparasites. Cytology of an impression smear from a pustule revealed neutrophils and acantholytic keratinocytes. Bacterial and fungal infections had been ruled out at the previous local veterinary clinic. The blood profile (complete blood count and serum biochemistry) showed leukocytosis [white blood cells (WBCs) 58100/μL] and mild anemia [packed cell volume (PCV) 27%]. Serum biochemistry revealed an elevation of liver enzymes [alkaline phosphatase (ALP) 1828 U/L and gamma glutamyl transferase (GGT) 22 U/L], most likely caused by the long-term corticosteroid treatment. Due to malnutrition, hypoproteinemia was also present [total protein (TP) 3.3 g/dL, albumin 2.2 g/dL].

**Figure 2:**
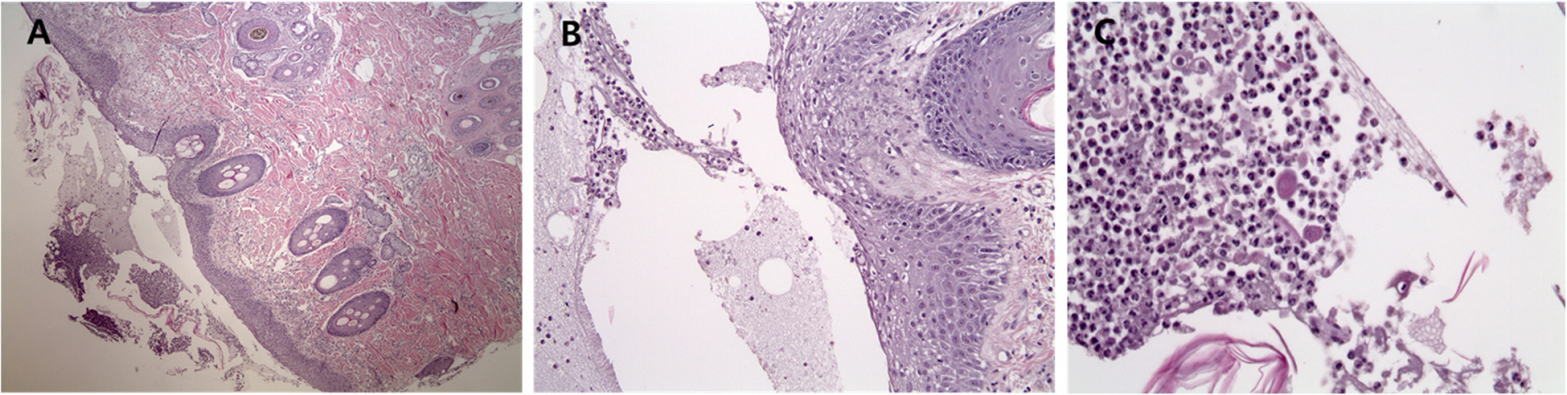
**Histopathology of a skin lesion.** Superficial crusting composed of a mixture of neutrophils, fibrin debris, and numerous acantholytic cells **(A)**, acanthotic epidermis **(B)**, perivascular, mild, mostly mastocytic and neutrophilic dermal inflammation **(C)**, and characteristic acantholytic cells. (H&E staining, magnification × 40, ×100, and × 200).

The definitive diagnosis of pemphigus foliaceus was made by histopathological examination of skin biopsies taken from the lesions on the forelimbs. Histopathology revealed a mixture of neutrophils, fibrin debris, and numerous acantholytic keratinocytes. The dermal inflammation was mild and mostly mastocytic and neutrophilic (Figure 2). The patient was treated with prednisolone (4 mg/kg/day, orally twice daily) for immunosuppression, cephalexin (60 mg/kg/day, orally twice daily) for controlling secondary infections, and with liver protectant drugs (lefotil 1 T/day, silymarin 20 mg/kg/day, and ursodeoxycholic acid 20 mg/kg/day, orally twice daily). The symptoms initially improved with a decrease in pruritus; however, recurred after one month. Cyclosporine (5 mg/kg/day, orally once daily) and azathioprine (2 mg/kg/day, orally once daily), were prescribed adjunctively but did not improve the clinical signs.

**Figure 3:**
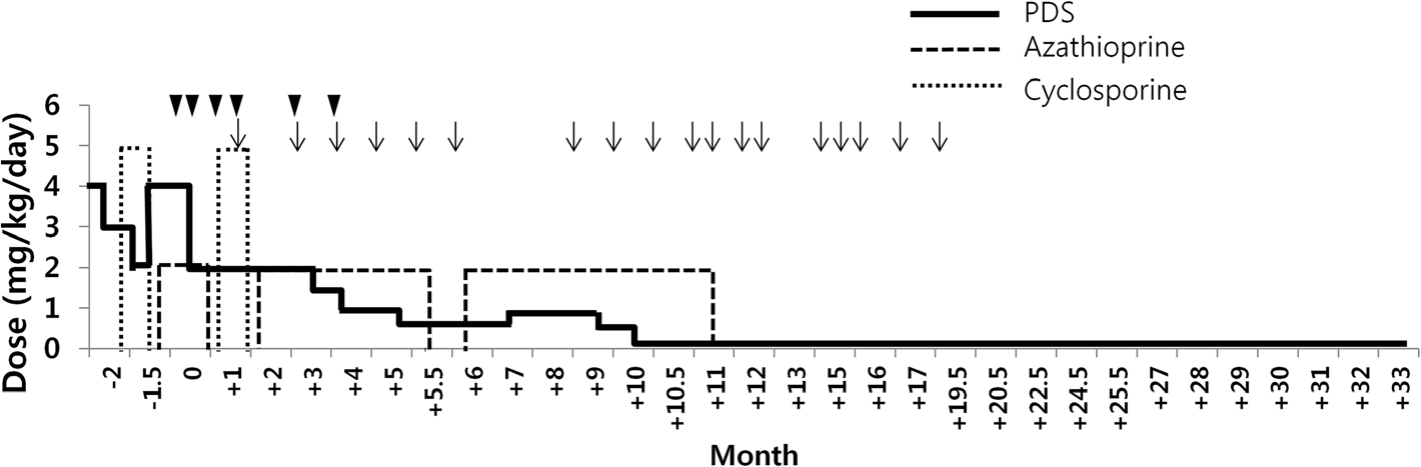
**Dosage changes of immunosuppressants after CTLA4 overexpressing ATMSCs (CTLA4-ATMSCs) and/or naive ATMSCs treatment.** The dose of prednisolone was gradually reduced and azathioprine was discontinued. Arrow heads indicate the time of CTLA4-ATMSCs administration and arrows indicate the time of naive ATMSCs administration.

**Figure 4:**
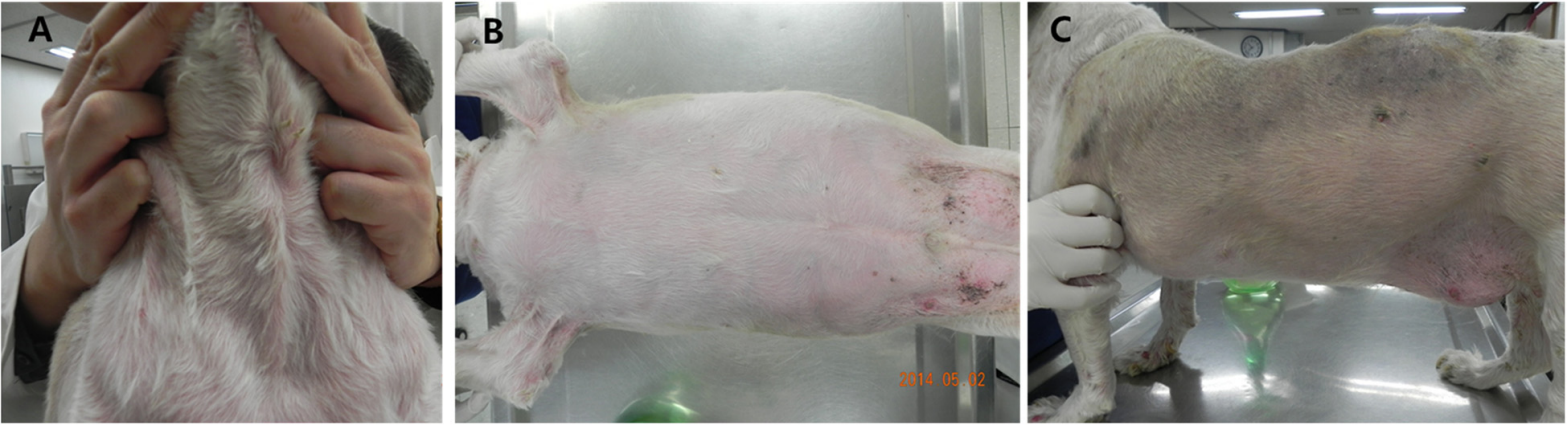
**Physical examination after CTLA4 overexpressing ATMSCs (CTLA4-ATMSCs) and/or naive ATMSCs treatment.** No lesions are present on the neck **(A)**, abdomen and inguinal area **(B)**, and dorsal region of the trunk **(C)** after stem cell therapy.

**Figure 5:**
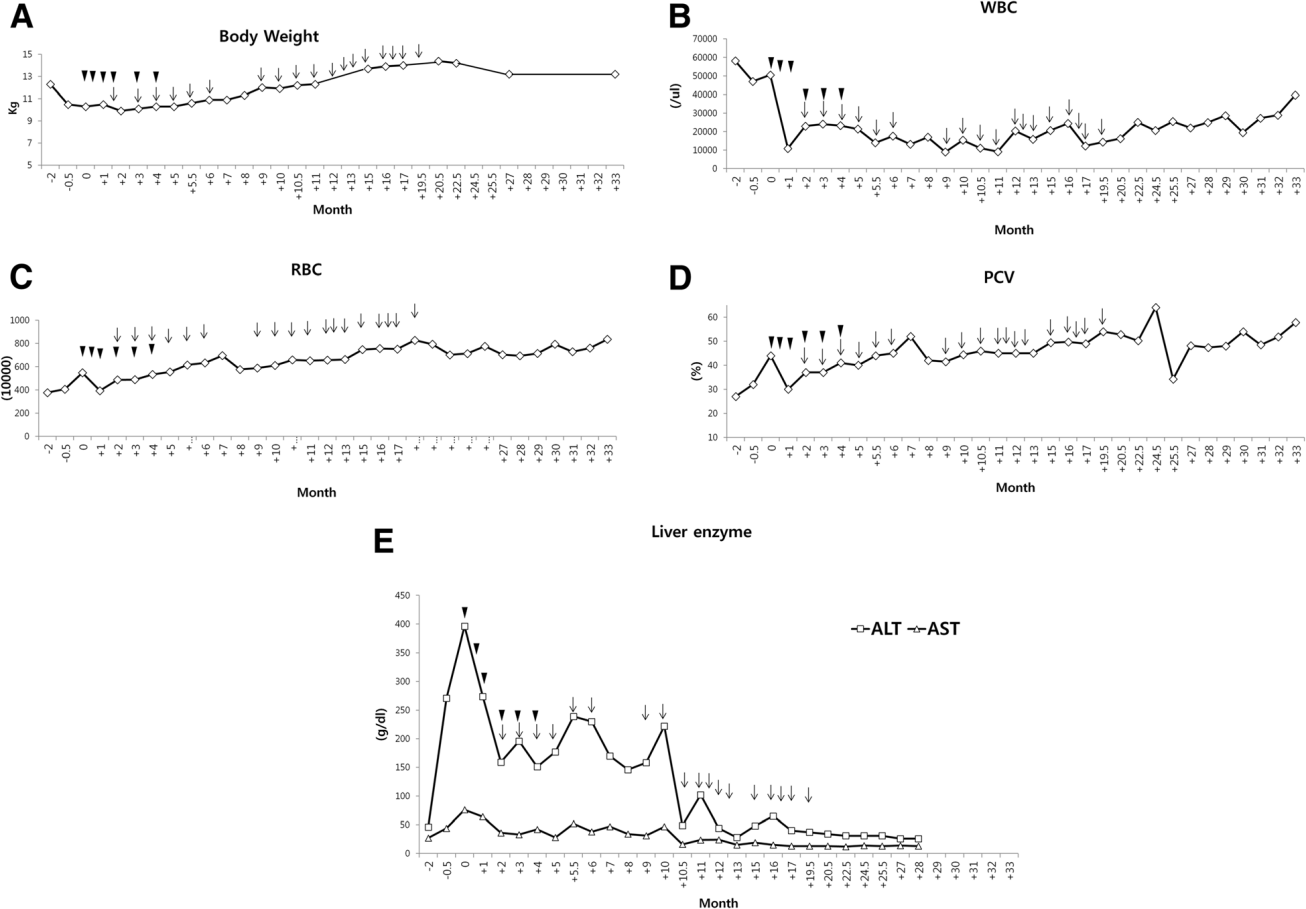
**Changes in body weight and blood profile after CTLA4 overexpressing ATMSCs (CTLA4-ATMSCs) and/or ATMSCs treatment.** An increase in body weight **(A)** a reduction in number of leukocytes **(B)**, an increase in the RBC count **(C)** and packed cell volume (PCV) **(D)** and a decrease in the liver enzyme concentration **(E)** are shown. Arrow heads indicate the time of CTLA4-ATMSCs administration and arrows indicate the time of naive ATMSCs administration.

Because none of the treatment had the desired effect, ATMSCs injection therapy was decided with the consent of owner. Stem cell therapies were carried out in the Cell Therapy and Animal Cloning Clinic of VMTH with the approval of College of Veterinary Medicine, Seoul National University. Canine ATMSCs was provided by K-STEMCELL Co. Ltd. (Seoul, Korea). To increase the potency, the dog *CTLA4* gene was transduced into canine ATMSCs by lentiviral expression systems as described in a previous study [15]. Canine ATMSCs and *CTLA4* overexpressing ATMSCs (CTLA4-ATMSCs) positively expressed CD29, CD44, and CD90, and did not express the surface markers CD34 and CD45. The patient received CTLA4 ATMSCs (2 × 10^6^ cells/kg) and/or ATMSCs (1 × 10^7^ cells/kg) intravenously. Additional medicinal treatments, antibiotics (cephalexin 60 mg/kg/day, orally twice daily) and with liver protectant drugs (lefotil 1 T/day, silymarin 20 mg/kg/day, and ursodeoxycholic acid 20 mg/kg/day, orally twice daily) were prescribed continually. After the first administration of CTLA4-ATMSCs, the dog became less pruritic, and the skin lesions improved. CTLA4-ATMSCs administration was performed 6 times and naive ATMSCs administration was performed 18 times over a period of 20 months at intervals of 2 to 8 week. CTLA4-ATMSCs and naive ATMSCs were concurrently transplanted at 4th, 5th and 6th administrations. During these 20 months, the dose of prednisolone was gradually reduced and azathioprine was discontinued (Figure 3). No recurrence of the skin lesions was seen (Figure 4). After the termination of ATMSCs treatment, the skin lesions were well managed with a low dose of prednisolone (0.25 mg/kg/day, orally once daily). Furthermore, the body weight increased and the blood profile showed an improvement in the leukocytosis, anemia, and liver enzyme elevation together with an improvement in the body condition (Figure 5). The manageable state lasted for 12 months after the last ATMSCs treatment. Unfortunately, this dog died newly developed pulmonary edema. At this time, skin lesions showed mild crusting which were manageable with low dose prednisolone.

## Discussion

The most common lesions of pemphigus foliaceus are scales, crusts, pustules, epidermal collarettes, erosions, and erythema. Ulcerations are occasionally observed indicating a deeper disease or secondary bacterial infection. In severe or chronic cases other observed signs include lymphadenopathy, edema, depression, fever, and lameness when the footpads are involved [16]. The initial skin lesions occur on the face and ears. The feet, claw beds, footpads, and groin are commonly involved and multifocal or generalized lesions develop within 6 months. Waxing and waning of the condition is common [17].

Autoimmune diseases are caused by an uncontrolled immune response against self-antigens. Immunosuppressive therapy is an important element in the treatment of pemphigus foliaceus [18]. Common immunosuppressive medication includes corticosteroids, cyclosporine, azathioprine, chlorambucil, cyclophosphamide and human immunoglobulin. In addition, management of autoimmune skin disease includes topical antimicrobial therapy and/or systemic treatment. Symptomatic treatment with gentle keratolytic shampoos such as chlorhexidine is often indicated and antibacterial and anti-yeast therapy should be prescribed where appropriate. These need to be continued until the disease is under control.

Although pemphigus foliaceus is considered a manageable chronic disease, the reported survival rate after 1 year is only 53% [19] and 25% of the dogs do not show complete remission with immunosuppressive therapy [20]. Because of the high incidence of side effects of corticosteroids and the large number of deaths within the first year of treatment, combination treatment with cyclosporine or azathioprine is suggested to decrease the maintenance dose of corticosteroids [8]. Combination therapy has improved clinical signs in some cases. However, cyclosporine absorption and clinical efficacy can vary markedly among patients and some may not respond to treatment despite attainment of target blood cyclosporine concentrations [21]. In addition, it has been reported that the combination of prednisolone with azathioprine can cause gastric ulceration in dogs [22]. Therefore, alternative treatments are required to substitute the immunosuppressive drug.

MSCs are largely studied as a new therapeutic tool in a number of clinical applications. Many studies indicate that MSCs possess an immunosuppressive function [11] that can be modulated by cytokines and toll-like receptor ligands [23]. These immunosuppressive properties have attractive therapeutic potential for treatment of autoimmune diseases [24-27]. The poor immunogenicity of MSCs demonstrated *in vitro* and *in vivo* favors the use of allogeneic MSCs in clinical conditions. Overexpression of the *CTLA4* gene can increase the immunosuppressive properties of ATMSCs by lowering autoantibodies in serum [15,25].

This case report describes the clinical application of CTLA4-ATMSCs and/or naive ATMSCs in steroid refractory pemphigus foliaceus. Initial treatment comprised immunosuppressive doses of prednisolone after the diagnosis of pemphigus foliaceus. Treatment with prednisolone alone did not have the desired effect and combinations with cyclosporine and azathioprine were prescribed, with no improvement in the clinical signs. Side effects of the immunosuppression included melena and anorexia and the skin condition deteriorated, with lesions spreading over the whole body. After the first administration of CTLA4-ATMSCs, the skin lesions improved. CLTA4-ATMSCs and/or naive ATMSCs were administered 21 times over a period of 20 months with an interval of 2 to 8 weeks. A tapering dose of prednisolone was given concurrently. After termination of ATMSC treatment, the skin lesions were well controlled with a low dose of prednisolone and remained under control for 12 months. To the best of our knowledge, this is the first report describing the application of ATMSCs to treat a canine autoimmune disease and manage clinical remission. Although additional studies are needed to investigate the clinical application of CTLA4-ATMSCs or naive ATMSCs further, in cases where several pharmaceutical trials have failed, CTLA4-ATMSCs or naive ATMSCs transplantation could be considered as an adjunctive therapy.

## Conclusion

CTLA4-ATMSCs or naive ATMSCs transplantation could be beneficial as adjunctive therapy in both the remission and management of lesions by canine pemphigus foliaceus in veterinary medicine.

## Abbreviations

ALP: Alkaline phosphatase
ATMSCs: Adipose tissue mesenchymal stem cell
CTLA4: Cytotoxic T-lymphocyte antigen 4
CTLA4-ATMSCs: CTLA4-overexpressing adipose tissue mesenchymal stem cells
GGT: Gamma-glutamyl transferase
MSCs: Mesenchymal stem cells
NK cells: Natural killer cells
PBMCs: Peripheral blood mononuclear cells
PCV: Packed cell volume
TP: Total protein
WBCs: White blood cells
